# Topics of Public Interest

**Published:** 1918-02

**Authors:** 


### TOPICS OF PUBLIC INTEREST

---

**Automobile Accidents.** In N. Y. State in 1917 837 persons were killed by automobiles, as against 729 in 1916 and 455 in 1912. Grade crossing accidents killed 98, as against 82 in 1916 and 122 in 1915.

---

**Sugar Consumption.** The Dept. of Agriculture estimates the net retention of sugar in this country, corresponding approximately to consumption, as follows: 1914, 3,925,801 long tons; 1915, 3,851,327; 1916, 3,553,733; 1917, 3,777,640. Considering the recent shortage and efforts to control hoarding, it appears that the actual consumption must have increased somewhat by the depletion of what was an abundant reserve. Whether this increase is proportionate to that of the population or not, is difficult to decide, but it is doubtful whether, since the falling off of immigration, the population has increased more than 1% per annum.

---

**The Salamanca Hospital** opened its new extension Dec. 28. The cost was $9,800 for building and $3,300 for furniture and equipment.

---

**Lease of Hotels and Sanitariums for Hospitals.** The Glen Springs at Watkins and the Jackson Health Resort at Dansville have been leased by the U. S. Government, and the International and Cataract Hotels at Niagara Falls are under consideration.

---

**British Casualties** for December included 1,145 officers and 14,805 enlisted men, total 15,950, killed. The wounded or missing included 3,342 officers and 60,335 enlisted men, total 63,677. It will be noted that the ratio of total losses to deaths is about 4:1, the experience of most earlier wars. It is impossible to calculate the proportionate losses, but if, as has been stated, England has 4 million men in the service, the deaths are much higher than has previously been stated, approximately 4:1,000 per year for the ages 20-30.

---

**Physical Defects in Those Subject to Draft.** Rejections are said to range from 30 to 70% for physical reasons, and experience of the past has shown that many have passed the examining boards who are later weeded out. This is a matter that should cause alarm—or explanation.

---

**The Will of Dr. Ramon Guiteras** provides for various philanthropies in sums of $5,000-$10,000. One of the most interesting bequests is that of $10,000 to a protege, Lenoncio Campuzana, providing he take the name Ramon Guiteras.

---

**Death of Obese Celebrity.** Jim Simon, a negro, died in Philadelphia Dec. 28. He weighed 800 pounds and was said to be the heaviest man in the world.

---

**Female Nurses,** long known in armies and given an official status as trained nurses in the Spanish War, are now introduced for the first time into the American Navy. They will be placed on the hospital ships Comfort and Mercy, formerly known as the Havana and the Saratoga of the Ward line.

---

**The J. N. Adam Memorial Hospital** at Perrysburg is to be enlarged at an expense of $15,000. About 60 patients are on the waiting list.

**The Livingston Co. Tuberculosis Hospital** will be erected at Geneseo at a cost of about $19,000, utilizing a site and certain buildings already owned by the county.

---

**Second Lieutenants in the U. S. Army** (not represented in the Medical Corps) will hereafter be recognized by a single shoulder bar on the uniform coat and a loop of braid on the overcoat sleeve, instead of leather puttees and certain differences in the tailoring of garments as heretofore. The insignia are exactly the same as for first lieutenant, but are of gold and brown, respectively, instead of silver or black. The analogy to the oak leaves of major and lieutenant colonel is followed.

---

**Products of U. S. 1917.**. Gasoline 70 million barrels as against 35 millions in 1914. Petroleum 300 million barrels. Coal 550 million tons. Corn 3 billion bushels (600 million over last year).

---

**Government Loans.** The attention of our readers is directed to the need of further investment in Liberty Bonds, as they are announced. The war cost is too enormous to be borne entirely on the pay as you enter plan and the Bonds afford a means of automatically adjusting the time and distribution of the cost as equably as possible. By affording also an absolutely safe investment, they prevent individual losses of capital, discourage the waste of energy of promotors and thus help to conserve the wherewithal with which the war must ultimately be paid for. For the very large proportion of persons who have so small savings that no safe investment except the bank is available and for the small number of persons who have great wealth but not the capacity or inclination to engage in active business, so that bonds of a low interest rate—at least on the purchase price—are about the only path open. Liberty Bonds at 4% involve no sacrifice at all.

War Savings Certificates are a slightly different form of government loan, still more convenient for providing for small investments and especially for those who have comparably little to invest at a time. They are, so to speak, automatic savings banks, paying 4% compounded quarterly. The certificates become obligations when 20 five-dollar stamps are attached. These stamps have a par value of $5 on Jan. 1, 1925, will be sold for $4.12 in Jan. 1918 and at an increase of 1 cent a month up to $4.23 for Dec. 1918. There will be abundant information as to where the stamps can be purchased. The certificates can be registered to insure against loss, at any post office down to the third class and will be non-taxable, except for inheritance and income.

---

**Industrial Casualties.** Geo. E. Barton, an authority on re-education after disability, states that 35,000 deaths and 700,-000 disabilities sufficiently severe to cause incapacity for over 4 weeks, occur in this country annually So far as can be estimated from British statistics, the deaths are about what would be expected in an army of a million in about six months.

---

**Hysteric Gaits.** The complaint of muscular and joint pains, usually with, sometimes without some form of limp, has always been a source of embarrassment to army surgeons. The various conditions popularly called rheumatism and even classified imperfectly and awaiting full elucidation by the medical profession, the results of exposure and of the various strains of drilling, minor accidents, etc., certainly do produce pain and disability of various parts of the body. For example a surgeon knows that he experienced such pain and disability himself for weeks, honestly feared that it would prevent him from assuming military service, but fortunately found that it disappeared after being exposed in a mitigated degree to the same hardships as a private. After such an experience, he cannot deny the genuineness of a complaint of backache or assert that a limp is assumed to escape military service. Even without personal experience, civil practice convinces all of the genuineness of similar troubles. Yet, as a military surgeon, one is equally certain that the number of cases complaining along similar lines includes many malingerers or those who have developed hysteria, past the degree of frank faking of a disability. In some cases, the gait is sufficiently characteristic to show that it has been assumed, either consciously or subconsciously. A genuine lameness is, as one neurologist well expressed it, simple and easy whereas an assumed gait is something bizarre and difficult. For example, one case—probably malingering only subconsciously—developed a gait that, at a distance suggested that an examining board had passed a man with congenital dislocation of a hip, and that, closely studied, especially in mounting a step, was an impossibly complicated projectile limp.

---

**Medical College Merge.** Bennett and the Chicago Colleges have united to form the medical department of Loyola University of Chicago, with Dr. Lawrence Ryan Dean and Dr. G. E. Wyneken Secretary.

**A Special Course for Health Officers** has been instituted at the University of Buffalo, including lectures on communicable diseases, milk and food inspection, etc. The health officers of western N. Y. are attending in gratifying numbers and will receive a certificate on the passing of an examination.

---

**Naval Hospital Construction.** Work has progressed to a considerable degree or has been completed, under an appropriation of four million dollars, providing for construction as follows: Portsmouth, N. H., 9 buildings; Newport, R. I., 13; League Island Navy Yard, 24; Norfolk, Va., 20; Charleston, S. C., 24; Pensacola, Fla., 24. Various other buildings are covered by an additional appropriation of 1½ million.

---

**Morbidity and Mortality Rates at Encampments in the U. S.** These show a most surprising range, from about 9:1000 to 167:1000 for weekly morbidity and from zero to 71 for the weekly deaths for a division of about 25,000 men. The rates are due mainly to pneumonia which, in spite of its well recognized status as an infection, seems to depend largely on exposure and insufficient clothing, although meningitis has in a few instances occurred as an epidemic but never to the degree of producing an excessively high morbidity or mortality rate for a division. Considerable local differences, not approaching the high rates, have also been due to a purely accidental concurrence of septicaemia, traumatic fatalities, and fluctuations in various infectious and other diseases, as might be expected in civil practice. Incidentally, it is well to remember that men of military age would have an annual death rate of about 5:1000 per annum in peace conditions, or about 2½ a week for a division of 25,000.

---

**Use of Grain for Distilled Spirits.** A recent government regulation limits distillers after Jan. 1, 1918, to the use of corn of inferior quality (below Federal Grade No. 6), except that malted barley or rye may be used for the conversion of starch.

---

**British Casualties,** for November, totaled 120,089; 1,152 deaths of officers and 24,292 of men; 3,537 wounded or missing officers, 91,108 men. It will be noted that, in spite of the changed conditions of warfare, we have almost the same ratio (1:4-1:5) between deaths and total casualties, as in past wars.

---

**The U. S. Marine Hospital** (Public Health Service) of Buffalo, accommodating 50 patients and thoroughly equipped for X-ray work, major surgery and refined diagnostic methods, has been extended in scope so as to be available for all branches of the federal service.

**Plans for a New Tuberculous Hospital** for Livingston County have been ordered. The buildings will be completed before the end of 1918 and will cost $19,000. The site is in Leicester, adjoining Genesee on the southwest and adjacent to the famous White Woman Spring.

**U. B. Squad in Navy Reserve.** Thirty-five students from the dental and medical department of the University of Buffalo have enlisted in the Naval Reserve. All of the recruits are of draft age.

**Children's Hospital Opened by Red Cross in France.** The hospital has 70 beds and is already overcrowded with diphtheria, measles, scarlet fever and whooping cough cases. In the first week of the American Dispensary over 2,400 children were examined, more than 1,900 being between 3 to 13 years.

**The Surgeon General** has recommended instruction of cooks, mess sergeants and mess officers in the National Guard Camps. Instruction will be given in food values, in balancing of meus and methods of cooking and serving meals.

**The Army Nurse Corps** now numbers 3,800. About 10 times this number will be required for an army of 1,500,000. The salaries are from $50 upward, with maintenance. Experience has already shown the superiority of trained female nurses over the male hospital corps. As soon as the nurses needed for active service are recruited, a reserve of 100 will be established with headquarters at Lakewood, N. J., where the Lakewood Hotel has been leased for use as a general hospital.

